# An Unprecedented Clinical Presentation due to Bacillus cereus Bacteremia in a Previously Healthy Adolescent

**DOI:** 10.7759/cureus.86920

**Published:** 2025-06-28

**Authors:** Filippos Filippatos, Vasiliki Karava, Konstantinos Kakleas, Adina Santou, Athanasios Michos

**Affiliations:** 1 First Department of Pediatrics, School of Medicine, National and Kapodistrian University of Athens, Agia Sofia Children's Hospital, Athens, GRC; 2 Infectious Diseases and Chemotherapy Research Laboratory, School of Medicine, National and Kapodistrian University of Athens, Agia Sofia Children's Hospital, Athens, GRC; 3 Nephrology, Agia Sofia Children's Hospital, Athens, GRC; 4 Allergy and Immunology, Agia Sofia Children's Hospital, Athens, GRC

**Keywords:** bacillus cereus, bacteremia, rice, seizures, teicoplanin

## Abstract

Febrile seizures are common among young children but unusual in adolescents. This report presents an exceptionally rare case of fever with seizures resulting from *Bacillus cereus* bacteremia in a previously healthy adolescent. Timely diagnosis and appropriate antibiotic treatment were critical for the patient’s recovery. A review of the relevant literature highlights the rarity and importance of recognizing systemic *Bacillus cereus* infections in adolescents. Systemic infections caused by *Bacillus cereus*, which are often linked to mild gastrointestinal diseases and febrile seizures, have rarely been documented in pediatric patients, except for neonates or immunocompromised individuals. This unique case highlights the critical importance of considering *Bacillus cereus* infection in the differential diagnosis of unusual fever and seizures in adolescents, particularly with recent dietary exposures like rice consumption.

## Introduction

Febrile seizures typically occur in early childhood, primarily between the ages of 6 months and 5 years, and are relatively uncommon in adolescents. *Bacillus cereus* is an aerobic or facultatively anaerobic, spore-forming, Gram-positive rod-shaped bacterium widely distributed in the environment, notably found in soil, dust, and on various foods, particularly rice and other starchy products [1]. It is closely related to other members of the *Bacillus cereus* group, including *Bacillus anthracis*, the causative agent of anthrax, highlighting its potential pathogenic capabilities [1]. *Bacillus cereus* is capable of surviving extreme environmental conditions due to its ability to form heat-resistant spores, enabling its survival in improperly cooked or stored food products. Upon germination under favorable conditions, *Bacillus cereus* produces two distinct groups of toxins: emetic toxin (cereulide) and enterotoxins, each causing different clinical presentations [1,2]. The heat-stable emetic toxin, cereulide, typically leads to rapid-onset nausea and vomiting within hours of ingestion. In contrast, heat-labile enterotoxins cause a diarrheal illness characterized by abdominal cramps and watery diarrhea, with the onset typically within 8 to 16 hours post-ingestion ​​​[1,2]. Given its environmental ubiquity, heat-resistant spores, potential for severe systemic infections, and emerging antibiotic resistance patterns, early recognition and prompt microbiological identification of *Bacillus cereus* are crucial in clinical practice to ensure appropriate therapeutic interventions and improve patient outcomes.

Systemic infections, including bacteremia and neurological complications, are rare in older children and adolescents and are usually documented in neonates or immunocompromised individuals [1,2]. Other invasive infections due to *Bacillus cereus* are relatively rare but can be severe, including bacteremia, endocarditis, meningitis, ocular infections, and severe wound infections [1,2]. Invasive infections have been associated with contaminated intravenous lines, surgical procedures, and trauma-related inoculation [1,2]. Antimicrobial resistance in *Bacillus cereus* isolates is increasingly being documented, particularly resistance to beta-lactam antibiotics such as ceftriaxone, complicating clinical management [1,2]. Hence, glycopeptide antibiotics, such as vancomycin and teicoplanin, are frequently preferred due to their reliable efficacy against resistant strains [1,2].

This case report emphasizes the rarity and clinical novelty of an otherwise healthy adolescent presenting with fever and seizures attributable to *Bacillus cereus* bacteremia, which, to our knowledge, is an event rarely documented in the medical literature.

## Case presentation

A previously healthy 13-year-old boy presented to the emergency department with a high-grade intermittent fever peaking at 39.2°C lasting two days, and a single episode of generalized tonic-clonic seizures, prompting the visit. The seizure duration was approximately three minutes, followed by a brief postictal phase without significant confusion, drowsiness, or neurological deficits. His medical history was unremarkable, with no previous seizure episodes, chronic illnesses, or neurological disorders. The patient was partially vaccinated and lived in a refugee camp with poor hygiene conditions. His dietary history notably included consumption of cooked rice approximately 24 hours prior to the symptom onset but without vomiting or diarrhea, prompting a suspicion for *Bacillus cereus* infection.

Upon presentation, his vital signs included a temperature of 38.8°C, heart rate of 102 beats per minute, respiratory rate of 18 breaths per minute, blood pressure of 110/70 mmHg, and oxygen saturation of 98% on room air. His physical examination was unremarkable, with no signs of meningismus or focal neurological deficits. Initial laboratory investigations showed a normal white blood cell count (8,700/mcL), hemoglobin at 13.5 g/dL, platelet count at 220,000/mcL, and C-reactive protein level at 0.8 mg/dL. Patient's laboratory data is presented in detail in Table 1.

**Table 1:** Detailed laboratory data WBC: white blood cell; CSF: cerebrospinal fluid

Parameters	Patient's Values	Reference Range
WBC count	8,700/mcL	4,000–11,000/mcL
Hemoglobin	13.5 g/dL	12.0–16.0 g/dL
Platelets	220,000/mcL	150,000–400,000/mcL
Phosphorus	4.2 mg/dL	4.0–6.0 mg/dL
Magnesium	2.3 mg/dL	1.5–2.3 mg/dL
Potassium	3.6 mmol/L	3.5–5.5 mmol/L
Sodium	138 mmol/L	135–150 mmol/L
Chloride	100 mmol/L	95–110 mmol/L
Calcium	10.1 mg/dL	8.2–11.0 mg/dL
C-reactive protein	0.8 mg/dL	<0.5 mg/dL
CSF WBC	0/mcL	0–5/mcL
CSF glucose	67 mg/dL	50–80 mg/dL
CSF protein	21 mg/dL	15–45 mg/dL

In this patient, the differential diagnosis included bacterial meningitis, viral encephalitis, metabolic disturbances (such as hypoglycemia, electrolyte imbalances), acute intoxication, primary seizure disorders (e.g., epilepsy), and, less commonly, systemic bacterial infections without overt meningitis, as observed with *Bacillus cereus* bacteremia. Given the patient's atypical age, absence of evident clinical signs such as meningismus, and recent dietary exposure, the possibility of an uncommon systemic infection prompted a comprehensive diagnostic work-up, including lumbar puncture, neuroimaging, and microbiological cultures, to effectively exclude other serious conditions.

Empirical intravenous ceftriaxone treatment (100 mg/kg/24h) was commenced, with pending microbiological cultures. Blood cultures drawn at admission were positive approximately eight hours after the seizure onset for *Bacillus cereus* resistant to ceftriaxone, prompting a switch to teicoplanin (100 mg/kg twice every 24h for the first three doses, followed by 100 mg/kg once every 24h). A comprehensive antibiogram for *Bacillus cereus* highlighting typical susceptibility patterns across various antibiotic classes is presented in Table 2.​​​​

**Table 2:** A comprehensive antibiogram for Bacillus cereus, highlighting typical susceptibility patterns across various antibiotic classes The bacterium demonstrates frequent intrinsic resistance to beta-lactam antibiotics, including ceftriaxone, primarily due to beta-lactamase enzyme production. Glycopeptides such as vancomycin and teicoplanin remain consistently effective and are recommended as first-line therapy for severe systemic infections. Aminoglycosides and fluoroquinolones generally exhibit good efficacy, though susceptibility testing is always advised due to occasional variability.

Antibiotic	Susceptibility
Penicillin	Resistant
Ampicillin	Resistant
Amoxicillin clavulanate	Resistant
Piperacillin-tazobactam	Resistant
Ceftriaxone	Resistant
Cefotaxime	Resistant
Cefepime	Resistant
Ceftazidime	Resistant
Meropenem	Susceptible
Imipenem	Susceptible
Vancomycin	Susceptible
Teicoplanin	Susceptible
Gentamicin	Susceptible
Amikacin	Susceptible
Tobramycin	Susceptible
Ciprofloxacin	Susceptible
Levofloxacin	Susceptible
Moxifloxacin	Susceptible
Erythromycin	Resistant
Azithromycin	Resistant
Clarithromycin	Resistant
Doxycycline	Susceptible
Minocycline	Susceptible
Tetracycline	Susceptible
Clindamycin	Resistant
Linezolid	Susceptible
Trimethoprim-sulfamethoxazole	Resistant
Metronidazole	Resistant
Colistin	Resistant
Rifampicin	Susceptible
Fosfomycin	Resistant
Nitrofurantoin	Resistant
Fusidic acid	Resistant

**Figure 1:**
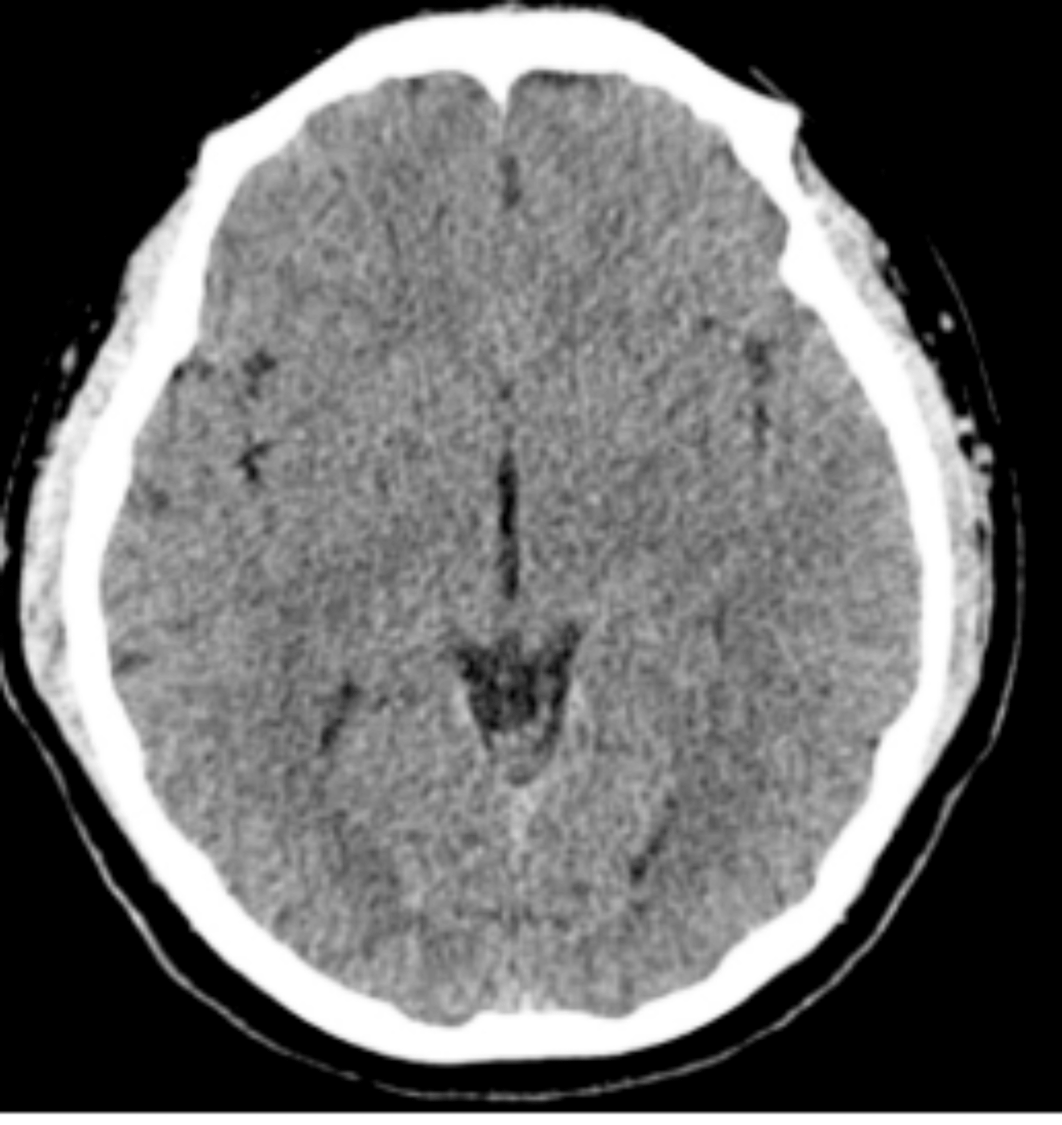
Computed tomography (CT) scan of the brain

Given the severity and atypical age for simultaneous fever and seizures, it was crucial to definitively exclude central nervous system (CNS) infection, especially bacterial meningitis; therefore, a computed tomography (CT) scan of the brain was essential before proceeding to lumbar puncture to avoid possible complications or contraindications. The CT scan performed urgently was unremarkable, excluding intracranial lesions or complications (Figure 1). The urgent lumbar puncture demonstrated normal cerebrospinal fluid (CSF) parameters: WBC count 0/mcL, glucose 67 mg/dL, and protein 21 mg/dL. Cultures from urine, feces, and CSF returned sterile (Table 1).

Repeat blood cultures confirmed persistent *Bacillus cereus* bacteremia after 48 hours. Following targeted antibiotic therapy initiation, the patient experienced rapid clinical improvement, became afebrile within 48 hours, and had no further seizure episodes. Teicoplanin levels were measured before the fourth administration dose and were normal (teicoplanin levels: 22 mg/L, normal values 15-30 mg/L). He was discharged on hospital day 5 in a stable condition, completing a 10-day course of teicoplanin therapy.

## Discussion

This case report underscores the exceptional rarity and novelty of *Bacillus cereus* bacteremia presenting with fever and seizures in an adolescent without underlying medical conditions. *Bacillus cereus* is a rod-shaped, aerobic, spore-forming bacterium known for causing gastrointestinal illness via contaminated food, especially cooked rice [3,4]. The organism produces heat-resistant spores and toxins responsible for vomiting and diarrheal syndromes, which are typically self-limiting [5]. Systemic manifestations, including bacteremia and neurological complications, are infrequent but can occur in neonates or immunocompromised patients and are often associated with high morbidity and mortality [6]. Systemic infections caused by *Bacillus cereus* are rarely described in healthy adolescents [7]. Neonatal infections often result from contaminated medical equipment, maternal colonization, or compromised immunity, leading to severe outcomes such as meningitis or sepsis [8]. Reports of adolescents experiencing systemic infections are scarce, and this case uniquely highlights bacteremia in an otherwise healthy individual without an underlying immunodeficiency or prior invasive procedures. A case report by Ikeda et al. highlighted clinical characteristics of adult bacteremia, emphasizing the variability and potential severity of *Bacillus cereus* bloodstream infections, though febrile seizures were not explicitly discussed [8].

The uniqueness of this case lies in the unexpected neurological presentation, generalized seizures with fever, without direct evidence of meningitis or cerebral involvement on imaging. The pathogenesis was likely toxin-mediated, as *Bacillus cereus* produces toxins capable of crossing the blood-brain barrier and triggering neurological symptoms [9]. However, explicit documentation of such presentations in adolescents remains sparse, reinforcing the case's novelty. Febrile seizures beyond early childhood warrant thorough investigation due to potential underlying infections, metabolic abnormalities, or neurological conditions. The exact mechanism by which *Bacillus cereus* induces seizures is not well understood but may involve bacterial toxins or inflammatory responses impacting cerebral function [9]. The absence of neurological abnormalities, rapid improvement with appropriate antibiotic therapy, and normalization of neuroimaging findings suggest toxin-mediated pathogenesis rather than direct bacterial invasion of CNS structures in this case.

A thorough literature review reinforces the rarity of adolescent febrile seizures associated with *Bacillus cereus* bacteremia [10]. A report identified a fatal family outbreak linked to contaminated food, underscoring the pathogenic potential of *Bacillus cereus* toxins and reinforcing the foodborne transmission route. Dierick et al. detailed epidemiological aspects of bacteremia in adults, further illustrating the clinical diversity and importance of prompt identification and management [11,12].

Studies by Lotte et al. and Tatara et al. comprehensively reported *Bacillus cereus* infections in neonates and immunocompromised individuals, highlighting severe disease outcomes and the necessity for heightened clinical awareness [5,9]. The findings corroborate our patient’s rapid recovery with proper antibiotic use, in contrast to the typically severe neonatal courses described elsewhere. The genetic basis for toxin production in *Bacillus cereus* infections has also been demonstrated, supporting the hypothesis that the toxin-mediated pathogenesis is potentially responsible for seizure induction [12,13]. Understanding these genetic mechanisms can aid in predicting clinical manifestations and guiding effective therapeutic strategies.

The isolation of *Bacillus cereus* resistant to ceftriaxone underscores the importance of antibiotic susceptibility testing to guide treatment. *Bacillus cereus* isolates often exhibit variable resistance profiles, necessitating careful antibiotic selection. Glycopeptides, such as teicoplanin and vancomycin, are usually effective against Gram-positive organisms, including resistant *Bacillus cereus* strains [14]. This case significantly broadens the recognized clinical spectrum of *Bacillus cereus* infections and highlights the importance of considering it in atypical febrile episodes in adolescents, particularly those with a recent consumption of rice or similar foods.

## Conclusions

This report describes an exceptionally rare and novel clinical presentation of *Bacillus cereus* bacteremia manifesting as febrile seizures in a healthy adolescent. Awareness and consideration of *Bacillus cereus* infections are crucial in unusual febrile seizure presentations, especially when dietary risk factors are present. Early identification and targeted antibiotic treatment ensure positive clinical outcomes in such cases.
